# Pedestrian Traffic Light Control with Crosswalk FMCW Radar and Group Tracking Algorithm

**DOI:** 10.3390/s22051754

**Published:** 2022-02-23

**Authors:** Peter Nimac, Andrej Krpič, Boštjan Batagelj, Andrej Gams

**Affiliations:** 1 Jozef Stefan Institute, Jamova cesta 39, 1000 Ljubljana, Slovenia; andrej.gams@ijs.si; 2 Jozef Stefan International Postgraduate School, Jamova cesta 39, 1000 Ljubljana, Slovenia; 3 Smart Com d.o.o., Brnčičeva ulica 45, 1231 Ljubljana, Slovenia; andrej.krpic@smart-com.si; 4 Faculty of Electrical Engineering, University of Ljubljana, Tržaška cesta 25, 1000 Ljubljana, Slovenia; bostjan.batagelj@fe.uni-lj.si; 5 Faculty of Maritime Studies and Transport, University of Ljubljana, Pot Pomorščakov 4, 6320 Portorož, Slovenia

**Keywords:** FMCW radar, smart traffic light, smart cities, smart mobility, traffic flow optimisation, point cloud, group tracking

## Abstract

The increased mobility requirements of modern lifestyles put more stress on existing traffic infrastructure, which causes reduced traffic flow, especially in peak traffic hours. This calls for new and advanced solutions in traffic flow regulation and management. One approach towards optimisation is a transition from static to dynamic traffic light intervals, especially in spots where pedestrian crossing cause stops in road traffic flow. In this paper, we propose a smart pedestrian traffic light triggering mechanism that uses a Frequency-modulated continuous-wave (FMCW) radar for pedestrian detection. Compared to, for example, camera-surveillance systems, radars have advantages in the ability to reliably detect pedestrians in low-visibility conditions and in maintaining privacy. Objects within a radar’s detection range are represented in a point cloud structure, in which pedestrians form clusters where they lose all identifiable features. Pedestrian detection and tracking are completed with a group tracking (GTRACK) algorithm that we modified to run on an external processor and not integrated into the used FMCW radar itself. The proposed prototype has been tested in multiple scenarios, where we focused on removing the call button from a conventional pedestrian traffic light. The prototype responded correctly in practically all cases by triggering the change in traffic signalization only when pedestrians were standing in the pavement area directly in front of the zebra crossing.

## 1. Introduction

The demands and expectations of transportation infrastructure users and the complexity of traffic regulation and control in modern cities are driving the need to include novel, advanced solutions into traffic flow optimisation and management [1,2,3]. All urban traffic optimisation and management depends on the feedback signal from sensors, while video-surveillance systems, coupled with autonomous artificial intelligence (AI)-driven decision algorithms, are being actively pursued [4,5], various solutions based on different sensors [6] are commonly applied for different categories of traffic participants.

Common examples, albeit for vehicles, are induction loop systems [7], which detect the disturbance of the loop’s own magnetic field by the presence of the vehicle’s metallic construction. Induction loop systems generally require a lengthy and complicated installation procedure, as pavement cutting is necessary for the installation [6,7]. For pedestrians, which are the focus of this paper, technologies that are seeing increasingly widespread use are the already mentioned video-surveillance traffic systems. These allow for the simultaneous and accurate traffic monitoring of several different traffic areas used by different traffic participants, and can also be quickly and accurately modified [6]. In general, this type of system is very cost-effective, especially in highly specialised cases, such as distinguishing between different traffic participants.

Despite the relatively simple installation, video-surveillance traffic systems still require frequent maintenance and lens cleaning. However, the main shortcoming of these systems is unreliable operation in low-visibility conditions, and novel approaches are being developed to handle such problems automatically [8]. Unreliable operation situations mainly occur during the night and in low-visibility weather conditions such as fog, rain and snow. Some video-surveillance traffic systems are even susceptible to incorrectly recognising shadows as traffic participants [6,9]. Furthermore, using cameras in public spaces brings up the question of privacy, primarily when these systems are used to monitor pedestrians [10,11]. To mitigate the privacy concerns caused by video surveillance, various techniques for privacy preservation have been developed [12,13,14], which is less than ideal because of the additional post-processing.

Radars share many advantages with video-surveillance traffic systems. They are just as capable of recognising various traffic participants and require similar installation procedures [7]. In contrast, radars are simpler to maintain; are not sensitive to reduced-visibility conditions; cannot invade privacy by design; and can very accurately determine the position, speed and direction of traffic participants within the field of view [15]. Different radars are already being used in ground traffic control, with the most common type being Continuous-Wave Doppler radar, which is generally only used for collecting speed data [6]. For other purposes, such as for measuring range or as a volume counting device, the Continuous-Wave Doppler radar is not accurate enough and not a suitable choice, as its signal lacks marking on a time axis [16]. Along with the Doppler-type radar, the second type of radar that is often used in ground traffic control is the Frequency-modulated continuous-wave (FMCW) radar, mainly used as a presence detector [7].

### Contribution of This Paper

**Figure 1:**
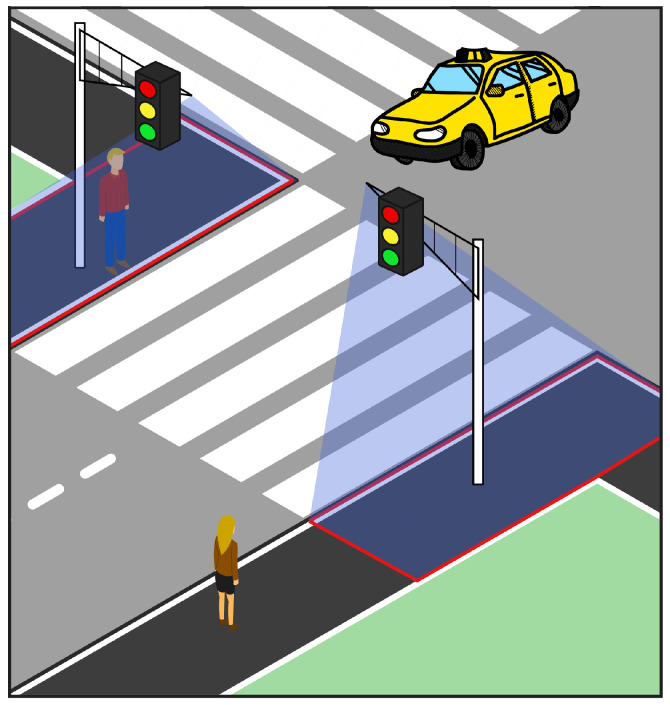
Crosswalk radar, placed in intersection, scanning for pedestrians with intention to cross the street. The observation area is marked with red rectangles.

In this paper, we demonstrate a proof-of-concept FMCW radar as an advanced form of pedestrian traffic light triggering mechanism. The proposed system uses a low-cost off-the-shelf FMCW radar as a kerbside detector which enables adaptive pedestrian crossing solutions with the support of multiple object tracking techniques. Figure 1 shows the suggested placement of a crosswalk radar with highlighted areas of interest, where pedestrians are detected. Our proposed solution relies on the group tracking GTRACK algorithm [17], which is used for multiple object tracking. The algorithm was modified to run on an external processor and can be further modified to work universally with similar FMCW radars. Additionally, we prepared a visualisation tool that shows tracked pedestrians in real time.

The first part of this paper describes a brief overview of existing technological solutions for human or pedestrian observation, detection and tracking. It also examines the shortcomings of these solutions and what kind of radar technology would serve as the best choice as an alternative to existing solutions. A short explanation of how FMCW radars work, an overview of the radar that we used and an explanation of how pedestrians are detected and tracked from radar measurements will be covered in this paper. The experimental process for evaluating the system’s performance, along with the experimental results, is described in the third part of this paper, which is followed by discussion and conclusions.

## 2. Related Work

### 2.1. Pedestrian Detection

Various sensors and techniques are currently being explored for detecting pedestrians among traffic participants. Pedestrian detection with computer vision technology remains an active research area and has improved significantly in recent years [18,19]. Automated pedestrian detection in traffic mainly relies on deep learning methods, which have shown consistently reliable operation [19].

**Figure 2:**
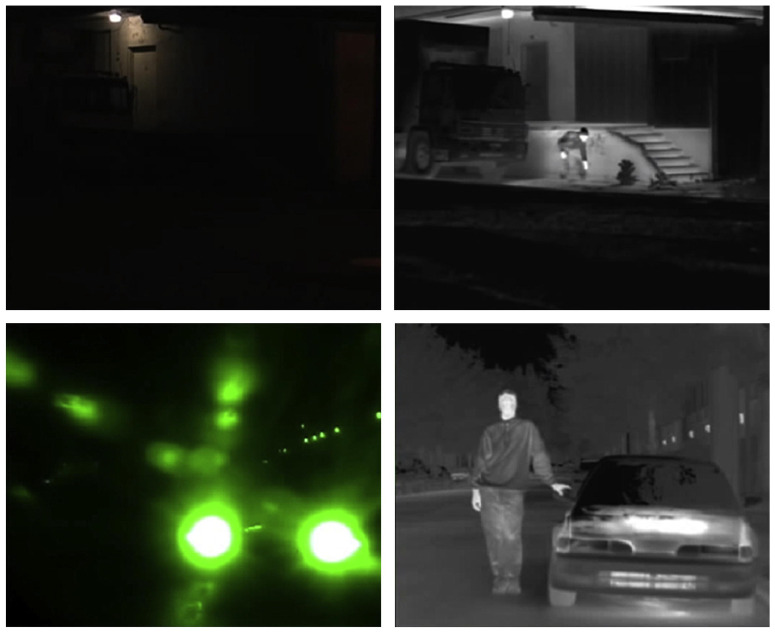
The left column shows images taken by a video camera and the right column show images of the same two scenes taken by an infrared camera. Reprinted/Adapted with permission from Ref. [21]. 2016 Elsevier.

Still, by the very nature of video cameras, such systems remain susceptible to reduced performance in low light conditions and false detections due to changing light levels. Larson et al. [20] have conducted an evaluation on pedestrian detection with optical sensors (video cameras) and thermal sensors (infrared cameras) and found that, in general, thermal sensors achieved higher detection accuracy than optical. As shown in Figure 2, the use of an infrared camera significantly improves visual detection in conditions where a regular video camera fails to do so. Additionally, the variability introduced by colour, texture, and complex background becomes trivial [21].

**Figure 3:**
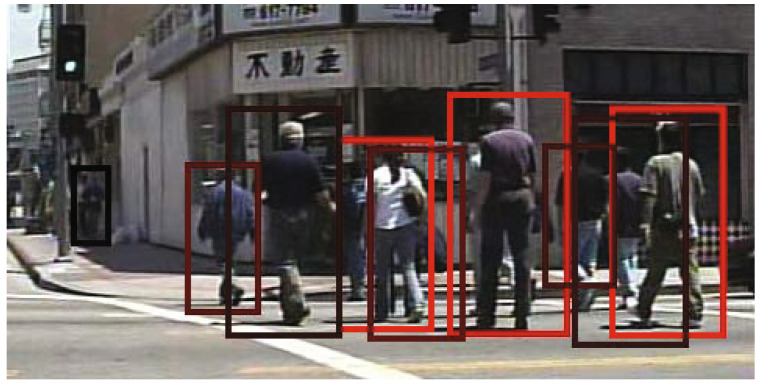
Example of pedestrian detection on an image from Caltech-USA test set by using a method SquaresChnFtrs. Reprinted/Adapted with permission from Ref. [18]. 2015 Springer Nature.

However, in Figure 2 we can also observe that pedestrians detected by an infrared camera lack shape, compared to those seen in Figure 3. Aside from this, other problems such as a low signal-to-noise ratio, low contrast and complex backgrounds hinder the reliability of infrared cameras without additional filtering [22].

Vision systems for pedestrian detection can use other types of sensors besides the aforementioned video and infrared cameras. Cheng [23] shows how point cloud information, obtained from an RGB camera, can be used to improve pedestrian detection. Lorente et al. [24] have shown that LIDAR and Time-of-flight (TOF) cameras can be used for point cloud acquisition and that such information can be, with the use of deep learning methods, applied for pedestrian detection. A millimeter-wave radar was used for a similar application, where Zhao et al. [25] proposed a point cloud classification algorithm for human–vehicle classification in advanced driver assistance systems (ADAS).

Aside from various imaging technologies, there are also examples of passive, non-imaging solutions for sensing a human presence. Examples are pressure-sensitive floor mats, which are not disadvantaged by the shortcomings of either video cameras or infrared cameras [9]. Compared to camera systems, these are more difficult to install or retrofit to existing areas and have seen only a limited install base, so their long-term reliability is yet to be proven [9]. Using floor mats to discern between different traffic participants or track their movements is still not reliable for large-scale application [26].

Apart from the use in traffic surveillance and management, as in  [27], automotive pedestrian detectors are actively being researched and improved upon for the use in autonomous vehicles. The applications vary from collision avoidance and advanced driver assistance systems [28,29,30] to detecting occluded and partially occluded pedestrians and other urban targets [31,32,33] to interactive autonomous vehicles [34,35].

### 2.2. Pedestrian Tracking Algorithms

For the reliable operation of traffic light controlling devices, participants need to be correctly identified and commonly need to have their position tracked within the observable area, mainly to avoid mis-triggering changes in traffic signalization [20].

For vision-based systems, several multiple object tracking (MOT) or multiple target tracking (MTT) approaches exist, which identify different objects in a video stream and then follow their trajectories [36]. Most modern algorithms in vision processing, working with either pixel information from cameras or point-clouds from depth cameras or radars, are based on some deep learning approaches [19,36]. Alternatively, classical algorithms with Extended Object Tracking (EOT) approaches [37,38,39] or GTRACK [17] can still be effectively applied. In this paper, we apply GTRACK to demonstrate the ability of the used FMCW radar. Other deep-learning-based tracking approaches could be used with the sensor data.

Generally, MOT approaches work according to the tracking-by-detection principle and employ either some or all of the following: steps [36]:

- Detection stage—input frame is analyzed and observed objects are identified within the input frame;
- Feature extraction/motion prediction stage—detected objects from the previous step are analyzed for their unique set of features. Motion prediction is an optional part of this step, where the algorithm predicts object’s approximate position in the next input frame;
- Affinity stage—features and motion predictions are used to compare presently detected objects with previously detected objects (from a previous frame or frames);
- Association stage—objects are associated with previously detected objects by assigning them the same ID and newly detected objects are assigned a new ID.

Each of these stages are performed by different kinds of algorithms and some algorithms perform more than one of these stages at once. In recent years, various deep learning techniques have been utilized in different stages of MOT. Different types of deep neural networks (DNN) are commonly applied for different stages, as shown in [36]. Optionally, MOT task can be extended with an additional segmentation stage. This approach is called Multiple object tracking and segmentation (MOTS). Voigtlaender et al. [40] have proposed a method for which they use TrackR-CNN to address detection, tracking and segmentation with a single convolutional neural network (CNN).

MOT can be divided into batch and online methods based on the task and the choice of these techniques (or the combination thereof) [36]. The difference is that batch methods can use future information for object tracking and can provide better tracking quality at the expense of real-time tracking. We are, therefore, required to use an online method for real-time pedestrian tracking, which can only use present and past information. Since deep learning algorithms are often computationally intensive, they are scarcely used in online MOT methods, though a few exceptions exist [36].

## 3. Radar for Pedestrian Detection

### 3.1. Types of RADARs

For accurate-enough tracking of pedestrians, we need to know pedestrians’ positions relative to the radar and the pedestrians’ walking velocities. The most suitable form of radar for this task is the FMCW radar, which has several advantages as opposed to other radar variations. The most common of them are Continuous-Wave Doppler radar (CW radar) and pulse-Doppler radar. CW radar continuously transmits a narrow-bandwidth signal, without interruption, at a fixed frequency. Signal reflection from a moving target will cause a shift in frequency due to a Doppler effect. The frequency shift directly corresponds to the target’s movement velocity; while this approach allows us to determine pedestrian’s movement velocity at any given moment easily, we cannot detect stationary pedestrians [9], nor can we discern pedestrian positions. This is because we cannot discern the round-trip time of the signal reflection since the signal is being transmitted continuously. The CW radar lacks necessary mechanisms, which could be used as a sort of timestamp. Without knowing when the signal was transmitted and later reflected, we cannot accurately determine the time delay between transmission and reception (round trip time τ). In general, round trip time, τ, is calculated by Equation [41,42]:

(1) $$\tau =\frac{2r}{c_{0}},$$

where *r* is the distance between a radar and a target and $c_{0}$ is the speed of light.

One solution is to interrupt the CW signal in repeated transmission intervals of equal duration, followed by typically longer intervals of radio silence. This type of radar is called a pulse-Doppler radar and enables the target’s position to be determined. This method presents a trade-off. Suppose we have long intervals of signal transmission without interruption (long pulse duration). In that case, the signal (pulse) will have more energy and will be able to traverse further distances and enable the detection of reflections from more distant targets. However, longer transmission times also increase the minimum detectable distance.To decrease the minimum detectable distance, we must, therefore, decrease pulse duration accordingly at a trade-off of decreased radar range, since short-duration pulses have less energy and dissipate more quickly [43]. Moreover, round trip times on very short distances are extremely difficult to measure accurately [9]. This difficulty increases with moving targets.

Alternatively, one can modulate the signal’s frequency of transmission rather than break it into pulses. This is called frequency-modulated continuous-wave (FMCW) radar. This type of radar transmits a radio signal in the form of a signal called chirp. In its simplest form, a chirp is a continuous wave signal, which linearly changes in frequency through the course of transmission. The radar we used in this paper is utilizing this type of chirp; more specifically an up-chirp, which increases in frequency over time.

The advantage of using an FMCW as opposed to CW radar or pulse-Doppler radar is that it can determine both: the target’s distance from the radar and its movement velocity. FMCW radar also has the following advantages [44]:

- Ability to detect targets on very short distances *r* (minimal range is comparable to the average wavelength of the transmitted signal);
- High accuracy in range estimation;
- Simultaneous transmission and reception;
- Signal processing after mixing stage is performed in a low-frequency spectrum, which simplifies the printed circuit board (PCB) design.

Hyun et al. [45] have used an FMCW radar for human–vehicle classification by using support vector machine (SVM) and binary decision tree (BDT) machine learning algorithms with very high classification decision rates. For this, they proposed three new Doppler-spectrum features, scattering point count (SPC), scattering point difference (SPD) and magnitude difference rate (MDR). Another recent use of FMCW radar was presented by Sang et al. [46], where they proposed a new method for direction-of-arrival (DoA) estimation in an autonomous driving application. They proposed an alternative to multiple signal classification (MUSIC), an established algorithm for DoA estimation, and took a deep-learning-based approach, where they trained a 3D convolutional neural network (3D-CNN) for DoA estimation. Kim et al. [47] have also recently proposed a new high-resolution and low-complexity estimation algorithm for FMCW radars. They proposed using a 2D Fast Fourier transform (2D FFT) algorithm for initial range and DoA estimation, with the purpose to reduce the search area of the pseudo-spectrum. Data from this reduced search area is then used as an input for the MUSIC algorithm to achieve higher resolution.

FMCW radars have also been proposed for pedestrian detection. For example, Rizik et al. [48] have demonstrated the use of 24 GHz FMCW radar for security gate monitoring. Their prototype used radar in conjunction with a Raspberry Pi computer for data acquisition, which was then forwarded to a remote personal computer (PC) for detection, recognition and target tracking. In this paper, we propose the using 60 GHz FMCW radar for the task of traffic-light triggering, with signal processing on a remote PC. The radar’s features are presented next.

### 3.2. FMCW Radar for Pedestrian Detection

**Figure 4:**
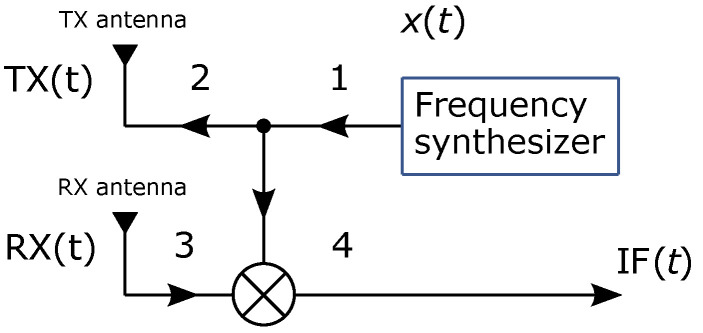
Simple block diagram of a generic monostationary FMCW radar with a single transmitter and single receiver.

**Figure 5:**
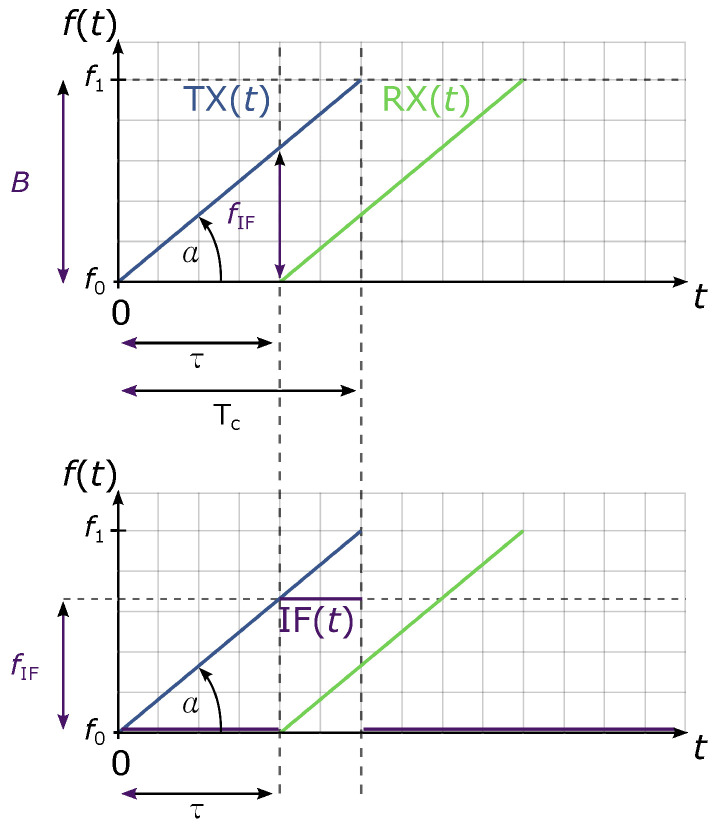
The intermediate frequency is the difference of frequencies of signals TX(t) and RX(t). $T_{c}$ marks the duration of a chirp, $f_{0}$ is the starting frequency and $f_{1}$ is the stop frequency.

To explain radar’s basic operating principle, Figure 4 shows the block diagram of a generic FMCW radar. First, the frequency synthesizer in step 1 periodically generates the chirp signal x(t)=TX(t), where TX stands for the transmitted signal. This is the signal that radar is constantly transmitting into space on a selected frequency range (step 2). The received signal RX(t)=x(t+τ), which is reflected at the target within radar’s range $r_{max}$, is received by the radar with a certain delay because of round trip time τ in step 3. Since the delay directly correlates to the distance *r* between the radar and the target, it also means that further-away targets will cause longer delays in the reception of a reflected signal. With FMCW radars, the time delay is not measured but is rather calculated from an intermediate frequency IF(t). The intermediate frequency is a constant continuous wave signal, which results from frequency mixing of the signals TX(t) and its own delayed copy RX(t+τ). In Figure 5, the height of intermediate frequency is directly correlated with the time delay τ, where more distant targets with longer time delays produce higher intermediate frequencies.

The height of the intermediate frequency $f_{\mathrm{IF}}$ is described by the Equation

(2) $$f_{\mathrm{IF}}\left(\tau \right)=\tan\alpha \;\cdot \;\tau =\tan\alpha \;\cdot \;2\;\frac{r}{c_{0}}$$

and tanα, with units [Hz/s], is the slope of a chirp, defined as

(3) $$\tan\alpha =\frac{f_{1}-f_{0}}{T_{c}}=\frac{B}{T_{c}},$$

where *B* is the chirp’s bandwidth.

The general range of radar *r* is described with the radar Equation [49], which describes the received power $P_{\mathrm{RX}}$ of the reflected radar signal at a distance *r* away from the target with

(4) PRX=PTXGTX1︸transmission·14πr2︸propagation︸powerflowofincidentwaves·1σ1︸reflection·14πr2︸propagation︸powerflowofscatteredwaves·HRX,eff1︸reception.

Power flow of incident waves is described by transmitted power $P_{\mathrm{TX}}$, multiplied with the gain of the TX antenna $G_{\mathrm{TX}}$ and with the factor of propagation in incident direction, from the antenna, directly towards the target. The radar cross-section of the target σ is the effective surface of the target, from which the incident propagation is reflected back towards the radar. The power flow of scattered propagation is then again reduced by the same factor of propagation. The final received power $P_{\mathrm{RX}}$ is multiplied by the factor of effective aperture (area) $H_{\mathrm{RX},\mathrm{eff}}$ of the receiving antenna. Effective aperture $H_{\mathrm{RX},\mathrm{eff}}$ can be expressed as

(5) $$H_{\mathrm{RX},\mathrm{eff}}=\frac{G_{\mathrm{RX}}\;\lambda^{2}}{4\pi }.$$

**Figure 6:**
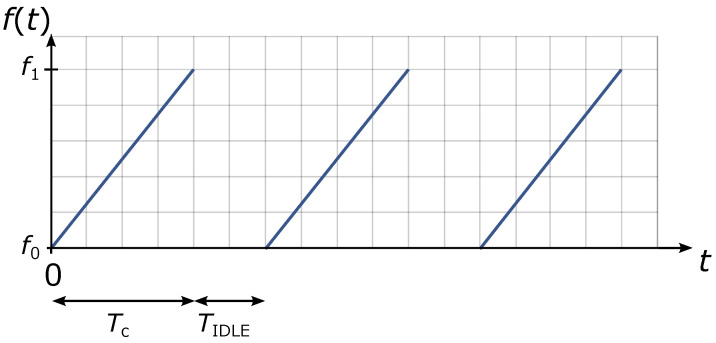
Idle time of the transmitter.

FMCW radar’s range is additionally limited by chirp’s duration $T_{c}$. Targets out of FMCW radar’s unambiguous range cause reflections with a delay of $\tau >T_{c}$. Those targets are ambiguous because they would appear as if they were closer to the radar. If needed, FMCW radar’s unambiguous range can be increased by increasing the chirp’s duration time $T_{c}$. Targets that are just slightly out of radar’s unambiguous range are mostly filtered during transmitter idle time $T_{\mathrm{IDLE}}$ as seen in Figure 6. $T_{\mathrm{IDLE}}$ is also the time when frequency synthesizer is reset to the starting frequency $f_{0}$.

### 3.3. mmWave Module IWR6843AOP

**Figure 7:**
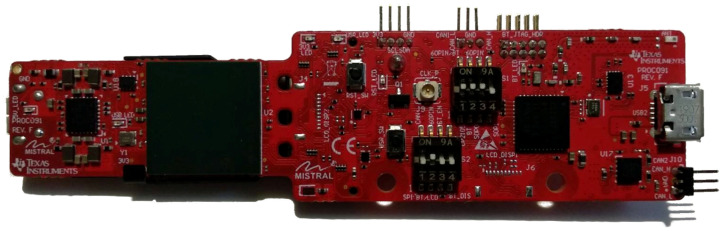
Evaluation module IWR6843AOPEVM (Rev. F), photographed from the front side.

In our experiments for pedestrian detection, we used an evaluation module IWR6843AOPEVM, by Texas Instruments (shown in Figure 7). The module is intended to test out the sensor IWR6843AOP, which operates in the millimeter-wave spectrum at frequencies ranging from 60 GHz to 64 GHz. The sensor is one of several in the family of radar-on-chip devices and is the first version of the integrated circuit with antennae on package, hence the suffix AOP. This particular characteristic makes this sensor a good choice for faster and easier development of the final product in later stages of development. The rest of the sensors from this family use microstrip patch antennae, which are connected to the sensor over the circuit board.

#### 3.3.1. Transceiver Capabilities

Sensor IWR6843AOP can transmit with a maximum power of 10 dBm within its operating frequency range. Seven antennae are integrated on the chip’s package, just below the top surface, and have antenna gain *G* of approximately 5 dBi. Three of those antennae are used for signal transmission and the remaining four for signal reception. The sensor is capable of discerning multipath propagation by utilizing three virtual MIMO arrays for digital beamforming. Transmitters can operate in three different modes of operation. In the first mode of operation, transmitters are alternatingly powered with the voltage of 1.3 V, so only two of the transmitters transmit simultaneously. In the second mode of operation, all three transmitters are powered with the voltage of 1 V and simultaneously transmit the same signal at different phase shifts, which enables electronic beam steering [50].

**Figure 8:**
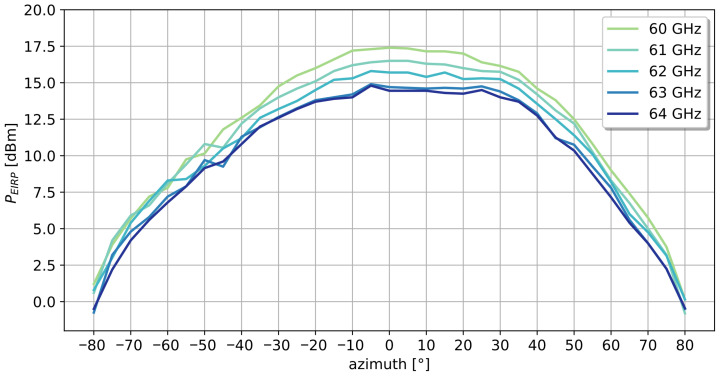
The approximate radiation pattern of TX2 transmitting antenna over azimuth at the best elevation angle.

By utilizing digital beam-forming techniques, all antennae provide a wide field of view over both azimuth (120°) and elevation (120°). Figure 8, based on data from [51], shows a radiation pattern of the TX2 transmitting antenna, a pattern that is similar to all the antennae on the used FMCW radar. The antenna is transmitting optimally at 60 GHz, and it has a beam width of 60° across the sensor’s operating frequency range. This radiation pattern is shared among all seven antennae. Each transmitter has effective isotropic radiating power of approximately $P_{\mathrm{EIRP}}\approx 15$ dBm.

From the transmitter’s effective isotropic radiative power, we can calculate a minimum safe distance, *r*, at which effective electric field falls below $|S_{\mathrm{eff}}|\leq 10\;W_{\mathrm{eff}}/m^{2}$ or below $|E_{\mathrm{eff}}|\leq 61\;V_{\mathrm{eff}}/m$. At the time of writing this paper, this is the value suggested by the International Commission on Non-Ionizing Radiation Protection (ICNIRP) as a minimum safe distance for the general public [52]. This distance also takes electromagnetic compatibility with other electronic devices into consideration, as they could start to function incorrectly in the presence of strong electromagnetic fields. According to Equation [50]

(6) $$r\geq \sqrt{\frac{Z_{0}\;P_{\mathrm{EIRP}}}{4\pi |E_{\mathrm{eff}}|}},$$

where $Z_{0}=377\;\Omega$ (free space impedance), the minimum safety distance is r=12.5 cm. The European Commission [53] stipulates that electronic devices with $P_{\mathrm{EIRP}}\leq 10$ W should be mounted at the height at least 2.2 m above general public walkway to ensure a distance of at least 20 cm between the main antenna lobe and a 2 m-tall person, by citing the IEC standard EN 62232:2017 [54].

#### 3.3.2. Chirp Configuration Parameters

For pedestrian detection, the radar was configured for a maximum detectable range of 10.95 m with a range resolution of 21.4 cm. The reason for such a short range of detection is because, in our study, we are only interested in the immediate pavement area right in front of the crossing. More precise range resolution is also of lesser importance, since we are not trying to obtain the exact positions of pedestrians. It is only necessary to see whether the pedestrian is within the observable area and their velocity. For better detection, it is better to have a more precise radial velocity resolution as pedestrians and other targets will all have different movement velocities and are, thus, easier to separate from one another. Additionally, better velocity resolution helps to better separate stationary clutter from pedestrians who are standing still, while these pedestrians are not moving around, they do not remain completely motionless. Furthermore, most targets will not approach the radar in the radial direction but under different angles of approach, which reduces their radial velocity compared to their actual movement velocity. Because of this, it is favored to have a better resolution. The radar was, thus, configured for the maximum radial velocity of 5.12 m/s and radial velocity resolution of 0.08 m/s. Parameters that we used in our set-up are listed in Table 1.

#### 3.3.3. Point Cloud

In each time instance, the radar measures reflections and combines them into a point cloud. Points in the cloud form clusters, which represent different targets within the sensor’s range.

**Figure 9:**
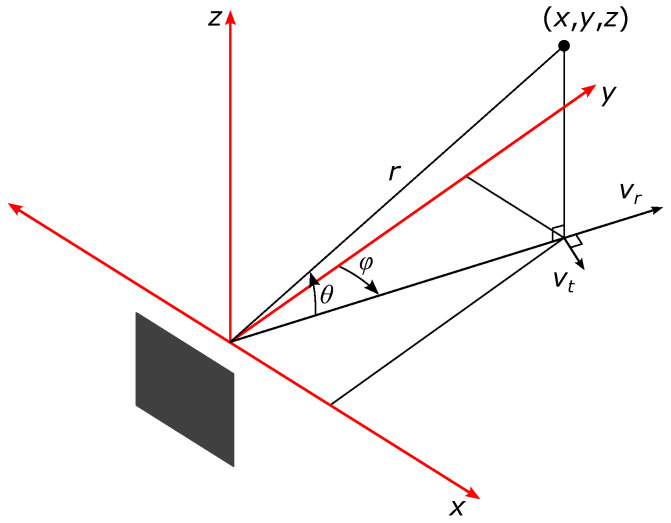
Representation of a single point of reflection in the sensor’s coordinate system. Distances *x*, *y* and *z* describe point’s position in radar’s Cartesian space; radius *r*, elevation angle θ and azimuth angle φ describe point’s position in spherical coordinates; point’s radial velocity is represented by $v_{r}$, and tangential velocity is represented by $v_{t}$ (the latter is not measured by the radar).

Each point in the cloud is described with the following parameters:

- position in Cartesian space, relative to sensor’s position as shown in Figure 9;
- radial velocity $v_{r}$;
- signal to noise ratio of reflected signal;
- noise level.

## 4. Pedestrian Detection and Tracking with GTRACK

The detection and tracking of pedestrians are performed by the group tracking algorithm GTRACK [17]. GTRACK was initially developed by Texas instruments to be used with their line of mmWave sensors. Since the algorithm was designed to run on the sensors’ integrated processor, we modified it into a python module, so it can run on any external CPU capable of running python. By doing so, we off-loaded the detection and tracking off of the integrated processor. Off-loading the detection and tracking from the integrated processor allows the sensor to process more reflection points while still meeting high real-time requirements. GTRACK was modified with the idea that it can be used alongside the out-of-the-box firmware, which comes pre-flashed on off-the-shelf mmWave FMCW radar. Since GTRACK uses point cloud data as the main input, it can be further modified to work with different sensors, not necessarily with an FMCW radar. This especially benefits sensors with limited processing capabilities and can only output measurements. Nevertheless, ideally, the same device would perform measurement acquisition, detection, and tracking.

GTRACK takes the point cloud data as the input, which is then processed in several steps as spatial filtering in the form of clustering and temporal filtering in the form of tracking. The first step is the prediction step, in which the algorithm estimates the present position of each currently tracked object at time instance *n*. This step is completed by considering the centroid position of the object’s cluster from the previously known position in time instance n−1.

Next are the association and allocation steps, when clusters in the point cloud data are associated with either one of the currently tracked objects’ track. In the case of a newly detected object, a new unique track is allocated. In the association step, a gate is formed around each predicted centroid. Measurements within the gate are then associated with the nearest existing track.

**Figure 10:**
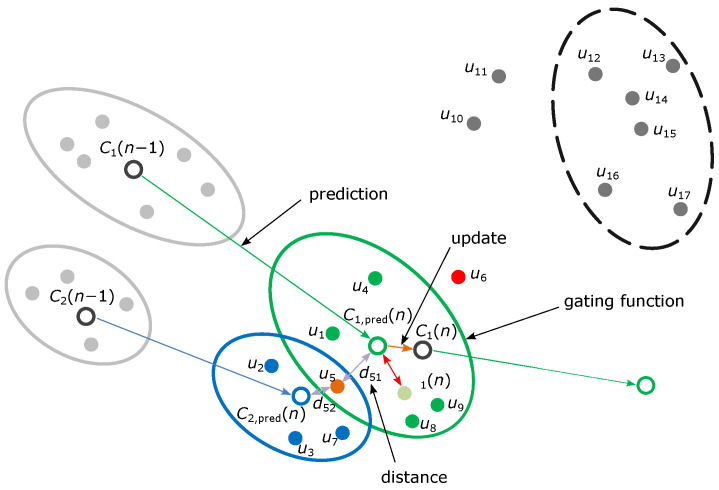
Single step of GTRACK algorithm.

If any measurements remain unassociated, new tracks are created, associated with clusters of measurements that remained after the association step. This process is similar to DBSCAN clustering [17] but only completed for unassociated measurements. Measurements are clustered together in the order of closest velocity, then closest distance. A new tracking object is initialized if a cluster contains enough measurement points with a strong enough combined signal-to-noise ratio (SNR). The described process is shown in Figure 10.

For each different kind of object we want to track, we must initialise a separate GTRACK instance. Each instance contains the general description of the object type, e.g., pedestrian, cyclist, car, or any other traffic participant. For pedestrians, we initialise a GTRACK instance with parameters described in Table 2, Table 3 and Table 4. The parameters were determined empirically by scaling typical human dimensions and space requirements in Table 5. For depth limit and width limit, a space requirement for a person with an open umbrella was taken [55]; this also considers the space requirements for a person with walking crutches [56]. We equated both measurements because an umbrella is of a round shape. We also observed that measurement points scatter of a person without an umbrella was almost always of a cylindrical shape, irrelevant of persons’ orientation respective to the sensor. For the height limit, we considered the average height of an adult male (1.87 m) [57], that we empirically scaled to 2 m. This height is also closer to 1.92 m, as listed in [56]. The latter also lists the shoulder width of 99% of adult males at 0.52 m and abdomen width at 0.35 m.

Since a new instance of the GTRACK algorithm has to be run for each different type of object, it makes it additionally beneficial for it to run on an external processor. An external processor can more efficiently handle more concurrent instances than a sensor integrated processor, as the latter also has to manage measurement acquisition.

## 5. Experimental Evaluation

We designed an experiment with six different scenarios to evaluate pedestrian traffic light triggering. Each scenario was repeated 50-times. In the experiment we assumed that bypassing pedestrians would not remain in the radar’s observation area, and would exit this area quickly. Similarly, we assumed that pedestrians intending to cross the street would remain inside the observation area until they were given a green signal. Thus, in our prototype, the control of the traffic light was based on the time a person remained in the observation area. If a pedestrian remained within the area for a set amount of waiting time, the system would recognize this and act as if a pedestrian call button was pressed. We determined the waiting time before triggering a traffic light change empirically and set it to 10 s.

In the first scenario, participants entered and stood inside the observation area that represented the part of the sidewalk where pedestrians would wait for a green signal to cross the road. In each repetition, only one participant entered and was present in the observation area at a time. In this scenario, we observed how many times the system correctly recognized a waiting pedestrian and triggered the change in traffic signalization. If the system triggered a traffic signalization change, we counted that it responded correctly. If the system did not trigger a traffic signalization change, we counted it as an incorrect response.

In the second scenario, participants only passed by the observation area to check whether the system would correctly recognize that none of the detected pedestrians intends to cross the street and, therefore, should not trigger any change in traffic signalization. Again, in this scenario, only one participant was simultaneously present in the observation area at a time. If a green signal was given despite none of the participants stopping to cross the street, it would only disrupt traffic flow in a real-life scenario, which we counted as incorrect system response.

We also want to track and identify multiple pedestrians since multiple pedestrians may be concurrently present within the observable area. However, only once in a while do some of them stop to wait for a green signal. The latter was tested in the third scenario, in which two or more participants entered the observation area in quick succession, so there were always two or three participants present in the observation area at a time. Some participants left the observation area, and some remained inside the area. The participants only passing by the observation area should not confuse the system, which should still trigger traffic signalization changes for standing pedestrians. If the system triggered a traffic signalization change, we counted that it responded correctly. If the system did not trigger a change in traffic signalization while a participant was waiting for a green signal, we counted this as an incorrect response.

In the fourth scenario, two or three participants entered the observation area and immediately left it as if they had only passed by the street crossing. This behaviour should not confuse the system to falsely trigger traffic signalization changes as none of the participants in this scenario stopped inside the observation area.

In the fifth and sixth scenarios, we repeated the first and second scenarios where one person under an open umbrella entered the observation area to check whether the system still correctly recognized them and responded to their intent, either to cross the road or pass by.

### 5.1. Experimental Setup

**Figure 11:**
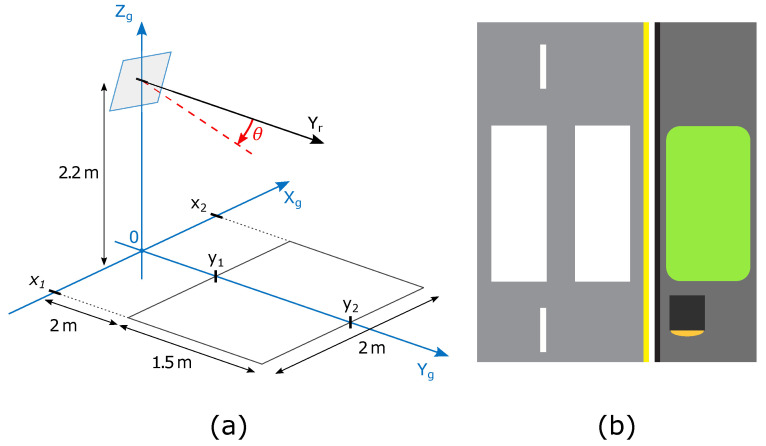
(**a**) Experimental setup. (**b**) Illustration of observation area next to a zebra crossing.

**Figure 12:**
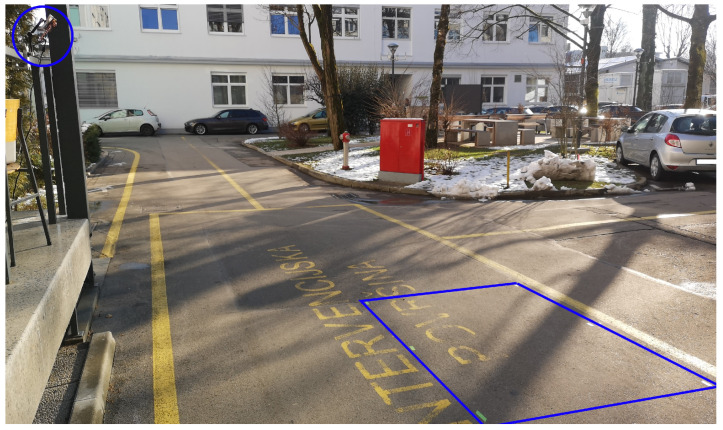
Photo of experimental setup.

For the experiment, we attached the radar on a vertical pole and set it at the height of h=2.2 m with an elevation tilt θ=26.5°. We arbitrarily set the observation area to be 1.5 m in length, 1.5 m wide and 2 m shifted away from the radar. We chose these measures to approximate an area in which pedestrians would stand to wait for the change in traffic signalization, where area length was chosen to be as long as an approximate width of a narrower zebra crossing Area width was chosen to approximate the width of a sidewalk, as shown in Figure 11 and Figure 12. The observation area is configured within the setup of the GTRACK algorithm and can be easily adapted to different situations.

To simulate walking on a sidewalk, participants in our experiment always entered by either of the two short edges, depending on the walking direction, and were moving in a tangential direction from the point of view of the radar. Participants who were passing by also exited the observation area by either of the short edges. In contrast, participants who stopped to cross the street exited the observation area by the longer edge as if it faced the zebra crossing.

### 5.2. Triggering Algorithm

For the experiment, we designed a simple algorithm that handles the GTRACK and it triggers changes in traffic signalization based on the GTRACK output data. On startup, our algorithm initiates the GTRACK algorithm and creates a table *tb_targets*. Table *tb_targets* keeps information for all currently tracked pedestrians along with the timestamps of pedestrians’ first detection. Upon detection, GTRACK assigns every pedestrian a unique identifier, which is used the whole time GTRACK maintains a lock on a pedestrian with that identifier. Pedestrians are stored in the table with their unique identifiers, which also serve as table indexing key. In the main program loop, at the beginning of each step, a current time is marked and stored in variable *timestamp*. Following that, GTRACK returns a list of detected pedestrians, which are then stored in the list *detected_targets*.

**Figure 13:**
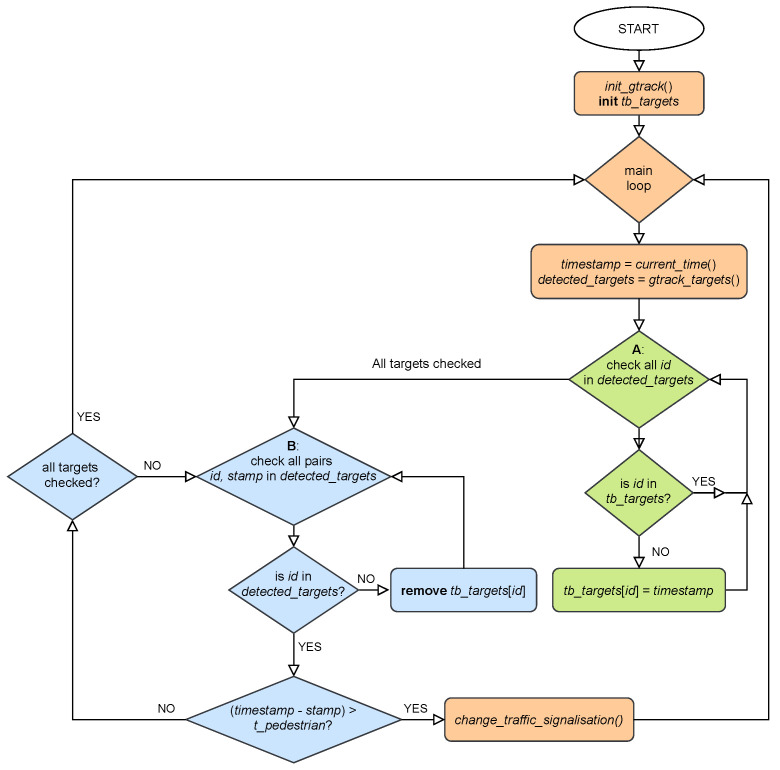
Flow diagram of pedestrian traffic light triggering algorithm.

Based on the presence and tracking of pedestrians in the observation area, our algorithm either responds to the elapsed waiting time by triggering a traffic signalization change and giving pedestrians a right of way. Alternatively, it keeps the pedestrian crossing closed and maintains an uninterrupted flow of road traffic if no individual pedestrian detains in the observation area for a given tracking time of 10 s. A more detailed description of the triggering algorithm is shown in the flow diagram in Figure 13 and explained in Algorithm 1.
**Algorithm 1** Pedestrian traffic light triggering algorithm**procedure**Traffic light control    **initiate** gtrack()    **create** tb_targets    **repeat**                      ▹Forever        **set** timestamp to current time        **get** detected_targets from gtrack()        **for all** detected_targets**do**:             ▹A           **if** target isnotintb_targets **then**:               **save** target and *timestamp* to tb_targets        **for all** targets **in** tb_targets**do**:            ▹B           **if** target isnotindetected_targets **then**:               **remove** target from tb_targets           **else**:               **if** target is in observable area longer than threshold time t≥10 s **then**:                   change traffic signalization    **until** shutdown

### 5.3. Results

Table 6 shows our experimental results. The second column shows the correct responses among all experimental repetitions for the given scenario. The third column shows the number of delayed responses of the system. The delayed responses are undesired, but still correct. Those are the cases where GTRACK temporarily lost lock on participants, which led to the reset of the tracking timer and, consequently, a longer waiting time. This column is only applicable to scenarios 1, 3 and 5. The fourth column shows the number of incorrect responses for the given testing scenario. During the experiment, we observed that the setup performed better when pedestrians had a higher radial velocity.

In almost all cases, pedestrians were still being detected, even if they had reduced radial velocity, for example, if they walked by the pedestrian crossing. It merely took more detection frames before GTRACK allocated clusters of observed pedestrians. This could easily be fixed with the below-proposed solutions. We could either elongate the walking strip, which represented the observable pavement area, or we could set the radar to face in the walking direction of pedestrians. The latter would also improve the detection of pedestrians obstructed by other pedestrians walking by their sides. However, this setup would, at the same time, obscure pedestrians who are walking in the same file. Nevertheless, pedestrians obstructed in this direction would possibly still be more easily detected since they would have better diversity in radial velocity.

In a few cases in scenarios one, three and five, GTRACK lost the lock on pedestrians because they stood too still. However, when they moved a little bit, GTRACK detected them again, which resulted in a delayed response of the traffic light triggering algorithm because the tracking timer restarted. In two cases in scenario three, the algorithm did not obtain a lock on a waiting pedestrian, as their radial velocity was not high enough for the GTRACK algorithm to detect them successfully. Furthermore, in one other case in the same scenario algorithm lost track of the standing pedestrian and did not recognize them again the second time. In some cases, in scenarios two and four, GTRACK did not lose lock after pedestrians left the observation area. This was due to when pedestrians moved too close to moving clutter when they exited the area, so the lock-on from pedestrians exiting the observation area was sometimes transferred to moving clutter. A similar error happened when a tracked pedestrian was exiting the observation area where another pedestrian entered, so exiting and entering pedestrians passed each other just at the edge of the area. In that case, the track from the exiting pedestrian was transferred to the entering pedestrian, which did not stop the tracking timer of the exiting pedestrian, nor did it start a new timer for entering pedestrian.

If we count all correct and delayed responses together as $n_{cd}=n_{c}+n_{d}=277+10=287$ and all incorrect as $n_{i}=13$ with a total of $n_{t}=300$, we obtained a system performance of $n_{cd}/n_{t}=0.9567$ or 95.67% and an error of $n_{i}/n_{t}=0.0433$ or 4.33%. However, if we count correct responses separately as $n_{c}=277$ and we count delayed responses along with the incorrect responses as $n_{id}=n_{i}+n_{d}=13+10=23$ with a total of $n_{t}$, we obtained a system performance of $n_{c}/n_{t}=0.9233$ or 92.33% and an error of $n_{id}/n_{t}=0.0767$ or 7.67%. Furthermore, if we were to exclude the delayed responses and count only correct $n_{c}$ and incorrect $n_{i}$ responses with a total of $n_{ci}=290$, we obtained a system performance of $n_{c}/n_{ci}=0.9552$ or 95.52% and an error of $n_{i}/n_{ci}=0.0448$ or 4.48%.

A separate evaluation of scenarios one, three and five shows, that the system correctly recognized a waiting pedestrian in $n_{cw}=137$ cases, combined with $n_{d}=10$ cases of system’s delayed response. With $n_{iw}=3$ incorrect responses over a total of 150 cases, we obtained a system performance of $(n_{cw}+n_{d})/150=0.98$ or 98% and an error of $n_{iw}/150=0.02$ or 2%.

If we similarly evaluate scenarios two, four and six, we can observe that system correctly disregarded pedestrians who were only passing in $n_{cp}=140$ cases and mis-triggered in $n_{ip}=10$ cases in a total of 150 cases. This gives us the performance of $n_{cp}/150=0.9333$ or 93.33% and an error of $n_{ip}/150=0.0667$ or 6.67%.

**Figure 14:**
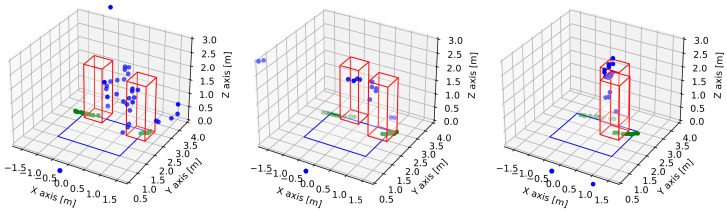
Two pedestrians in scenario four are being tracked by the GTRACK algorithm. Both pedestrians are entering the observation zone in opposite directions and are moving with higher tangential velocities than their radial velocities $v_{t}>v_{r}$.

**Figure 15:**
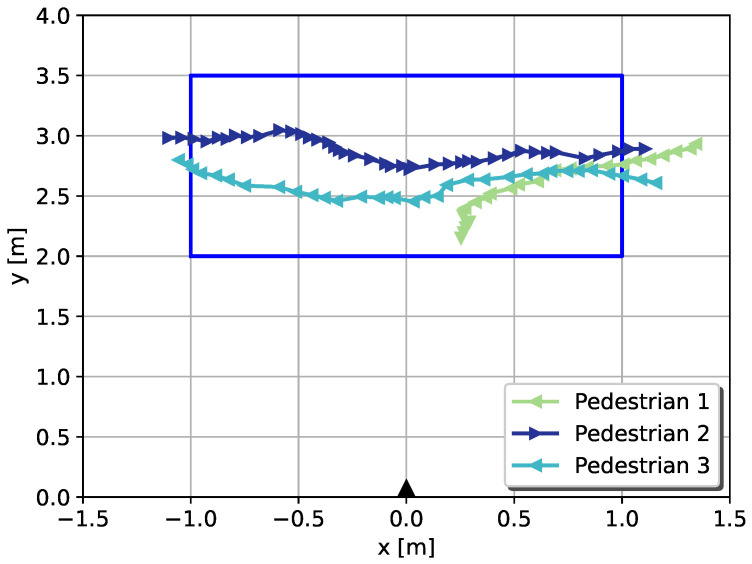
Example of three pedestrians’ tracks after they have been detected by the system. The track of pedestrian 1 is from the first scenario, and tracks of pedestrians 2 and 3 are from the fourth scenario. The radar’s position and orientation are marked with a black triangle.

Figure 14 shows an example of two pedestrians walking towards each other. Box frames represent the approximate calculated position of each pedestrian, green dots on the floor show previous locations of tracked pedestrians, and blue points are points of reflections detected by the radar. From these points, it is also impossible to recognize any identifiable features of pedestrians. An example of three separate pedestrians’ tracks is shown in Figure 15.

## 6. Discussion

Our results have shown that we were already able to detect and track pedestrians, along with their intent, by using a fairly simple algorithm. By using this as a basis, some more complex functionalities could be implemented even with the current setup. For example, a pedestrian call extension for pedestrians entering the radar’s observation area while the street is open for crossing. Though it is still better to have the observation area extended over a whole crosswalk for more reliable operation [20]. Our waiting pedestrian presence detection was also based on a fixed continuous observation time, which could be further studied as in [27]. These and other more complex functionalities can be implemented by using simple logic algorithms or perhaps training a neural network instead. Research in arrays of multistatic radar sensors, that are connected in a network [58], provides even more coverage and is opening new possibilities in advanced pedestrian tracking behaviors. This method additionally benefits by migrating GTRACK to a separated processor, as it would be easier to modify a single GTRACK instance to detect and track targets of multiple radars within the same multistatic configuration.

Besides logistical benefits, this method also has the potential to decrease traffic accidents involving pedestrians and, since this method also minimizes unnecessary vehicle stops [9], it can help to reduce the carbon footprint. An additional benefit of the proposed system is that it mitigates the need to touch the call button, which is especially important in times of epidemics, where touching a public surface might increase the possibility of infection. Using contactless detectors like one proposed in this paper or those described in [9,20,48], can contribute to slowing the spread of virulent diseases.

Since the proposed system is operating within an unlicensed radio frequency (RF) spectrum between 57 GHz and 71 GHz and with an average $P_{EIRP}<40$ dBm, it does not require any permissions from a regulator as, for example, Federal Communications Commission (FCC) [59]. Its operating power may need to be reduced to the average $P_{EIRP}\leq 10$ dBm to comply with the regulations. However, power requirements are set slightly differently, depending on the regional regulator of the RF spectrum. Additionally, since FMCW radars operate on different sweep frequencies, we do not expect to cause or suffer any interference from other FMCW radars, which is additionally beneficial for testing in a real-life scenario. To evaluate the system in a real-life scenario, we would thus need to acquire approvals from the local authorities, where testing would be conducted and from the operator of the experimental testing intersection.

## 7. Conclusions

In test scenarios, where we evaluated the performance of the proposed system for activating the green pedestrian signal, we have observed that the system responded correctly in 277 cases out of a total of 300 repetitions across all six experimentation scenarios. In 10 cases, the system’s response was delayed, but it still responded correctly for a total performance of 95.67% and an error of 4.33%. However, in the 10 cases where the system’s response was delayed, this was due to the system losing lock on a waiting pedestrian for a short time, leading to longer waiting times for those pedestrians. The system struggled most in cases where pedestrians arrived in strong tangential directions with low radial velocities. Pedestrians having low radial velocities then led to longer detection times. Compared to video-surveillance systems that either use a standard video camera or an infrared camera, this performance is constant through any lighting conditions. We want to point out that all of the experiments were performed in a dry weather environment. Therefore, the proposed system performance would have to be similarly evaluated in future studies, where the experiments would be performed in foggy and rainy weather.

Assessing different setups of radar position and observation areas is left for future research, the most interesting of which is using two radars to observe the same area. The system’s accuracy in positioning-detected targets is also yet to be evaluated. To do this, we need to use a system with known higher accuracy and one preferably not based on radar technology because, as we have observed, these radars struggle with targets moving in a tangential direction. An interdisciplinary study on the field of psychology may also be considered to find an optimal waiting time before the system triggers the change in traffic signalization.

To take full advantage of this design, we could extend the radar’s observation area over the whole crosswalk and continuing tracking while pedestrians have a green signal. This observation area extension makes it is possible to further optimize traffic flow by changing to a red signal only immediately after there are no more pedestrians crossing the street [9]. Furthermore, because observed pedestrians were moving in a tangential direction in respect to the radar, extending the observation area would allow the radar to face incoming pedestrians at a more favorable angle. We want to note that the radar can also be rotated in the azimuthal direction, which could, depending on the setup, also improve the radar’s detecting and tracking capabilities.

## Figures and Tables

**Table 1:** FMCW radar configuration parameters.

Parameter	Value
Starting frequency	60 GHz
Bandwidth	4 GHz
Slope	100 MHz/μs
Samples per chirp	64
Samples per frame	384
Max unambiguous range	10.95 m
Range resolution	21.40 cm
Max radial velocity	5.12 m/s
Radial velocity resolution	0.08 m/s
Measurement rate	15.00 Hz

**Table 2:** GTRACK gating parameters for pedestrian detection and tracking.

Parameter	Value
Depth limit	1.125 m
Width limit	1.125 m
Height limit	2.0 m
Doppler spread limit	0.7 m/s
Gain	3

**Table 3:** GTRACK allocation parameters for pedestrian detection.

Parameter	Value
Min SNR	30.0 dB
Min SNR obscured	160.0 dB
Min Velocity threshold	0.05 m/s
Min Points threshold	7
Max distance threshold	1.6 $m^{2}$
Max velocity threshold	2.0 m/s

**Table 4:** GTRACK state transition parameters for pedestrian detection and tracking.

Parameter	Value
det2actThre	6
det2freeThre	5
active2freeThre	20
static2freeThre	110
exit2freeThre	25
sleep2freeThre	600

**Table 5:** Typical human dimensions and space requirements [55].

Measurements	Value
Depth	1.125 m
Width	1.125 m
Height	1.87 m

**Table 6:** Experimental results of 300 repetitions across all six testing scenarios.

Scenario	Correct	Delayed	Incorrect
Scenario 1	47/50 (94%)	3/50 (6%)	0/50 (0%)
Scenario 2	46/50 (92%)	N/A	4/50 (8%)
Scenario 3	45/50 (90%)	2/50 (4%)	3/50 (6%)
Scenario 4	44/50 (88%)	N/A	6/50 (12%)
Scenario 5	45/50 (90%)	5/50 (10%)	0/50 (0%)
Scenario 6	50/50 (100%)	N/A	0/50 (0%)
